# Intrapelvic migration of a trial femoral head during direct anterior approach total hip arthroplasty: a case report and literature review

**DOI:** 10.3389/fsurg.2026.1872009

**Published:** 2026-07-22

**Authors:** Muxin Zhang, Longfeng Rao, Jing Shen, Weifeng Ji

**Affiliations:** The First School of Clinical Medicine, The First Affiliated Hospital of Zhejiang Chinese Medical University (Zhejiang Provincial Hospital of Chinese Medicine), Hangzhou, Zhejiang, China

**Keywords:** case report, direct anterior approach, intraoperative complication, intrapelvic migration, total hip arthroplasty, trial femoral head dislocation

## Abstract

**Introduction:**

The direct anterior approach (DAA) for total hip arthroplasty (THA) is increasingly adopted due to faster postoperative recovery. However, rare intraoperative complications such as intrapelvic migration of a trial femoral head remain poorly characterized, and optimal management strategies are not well established. This study aimed to describe a novel intraoperative retrieval technique and evaluate its feasibility and safety.

**Methods:**

We report a single case of intrapelvic trial femoral head migration occurring during primary THA performed via the DAA at our institution. Intraoperative findings, decision-making, and surgical management were documented. A modified approach involving partial transection of the rectus femoris muscle was employed to facilitate manual retrieval of the migrated component.

**Results:**

The migrated trial head was successfully retrieved manually after approximately 30 min. No laparotomy or additional surgical intervention was required, and no neurovascular or visceral injury was identified. The patient subsequently underwent postoperative rehabilitation, with the Harris Hip Score (HHS) was 77 points at discharge (week 4), and the latest HHS score was 83 point after 7-months follow-up (May 2026).

**Discussion:**

This case demonstrates that a minimally invasive, direct retrieval strategy may be a feasible alternative to conventional approaches such as laparotomy or laparoscopy, which carry higher risks of morbidity. The technique expands current management options for this rare complication and may help improve intraoperative safety. Further studies are needed to validate its generalizability and establish standardized treatment algorithms.

## Introduction

DAA is an effective surgical intervention for advanced osteoarthritis and other hip joint disorders, significantly improving patients' quality of life. However, intraoperative complications should not be overlooked. The reported overall complication rate of DAA is approximately 6.9%, including nerve injury, periprosthetic fracture, implant loosening, infection, and leg length discrepancy (1). Dislocation or migration of the trial femoral head is a rare but difficult-to-manage intraoperative complication. If left uncorrected, it may lead to pain, limited mobility, and even periprosthetic joint infection, adversely affecting postoperative recovery. Currently, the optimal management strategies for this complication remain controversial. In this article, we review the relevant literature and introduce a novel approach, aiming to provide an alternative strategy to manage intraoperative trial femoral head dislocation, especially for patients with moderate functional demands and intact preoperative quadriceps function.

## Case description

**Figure 1:**
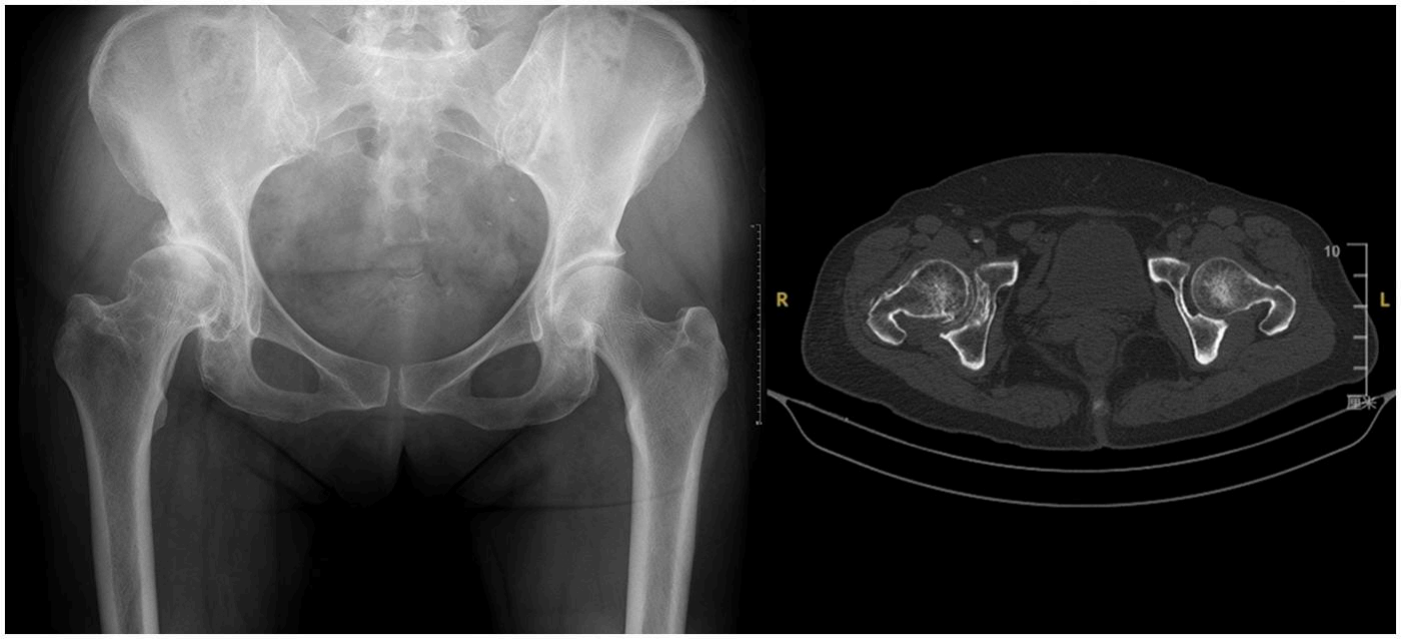
Anteroposterior x-ray showed the right femoral head has shifted outward and upward, with an irregular shape and irregular edges. There is also bone hyperplasia at the edge of the acetabulum.

A 70-year-old female had had right hip pain of insidious onset for over 3 years, which had progressively worsened over the past year and was particularly severe when climbing stairs, diagnosed with degenerative osteoarthrosis. Radiographic examinations and CT scan demonstrated right femoral head subluxation accompanied by bone hyperplasia (Figure 1). Physical examination revealed tenderness over the right hip and reduced range of motion. Her Harris Hip Score (HHS) was 68. The patient had the history of hypertension, taking valsartan orally to control blood pressure, without Glucocorticoids or any other drug used.

### Diagnostic assessment and therapeutic intervention

**Figure 2:**
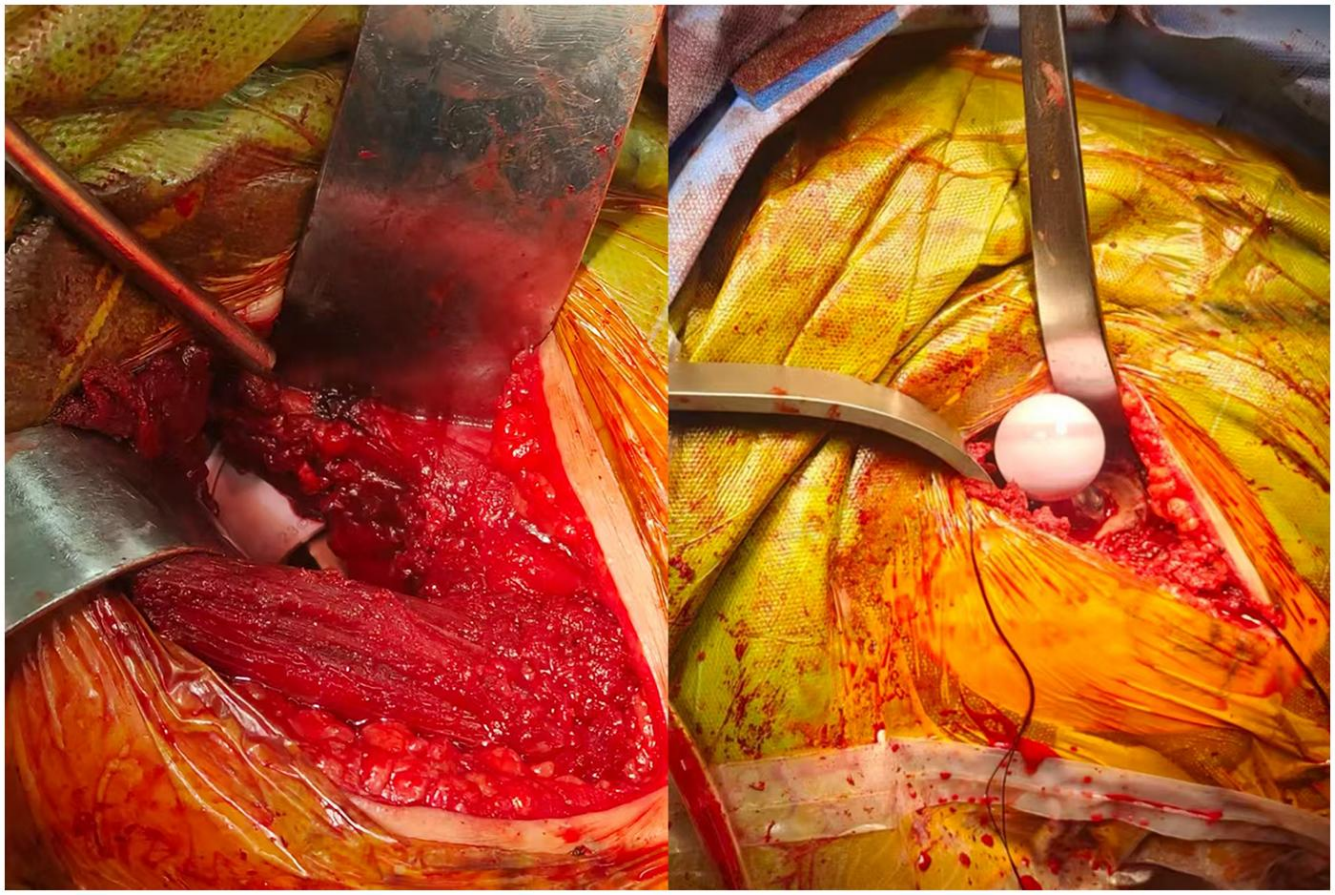
The trial head disengaged from the femoral stem during reduction.

**Figure 3:**
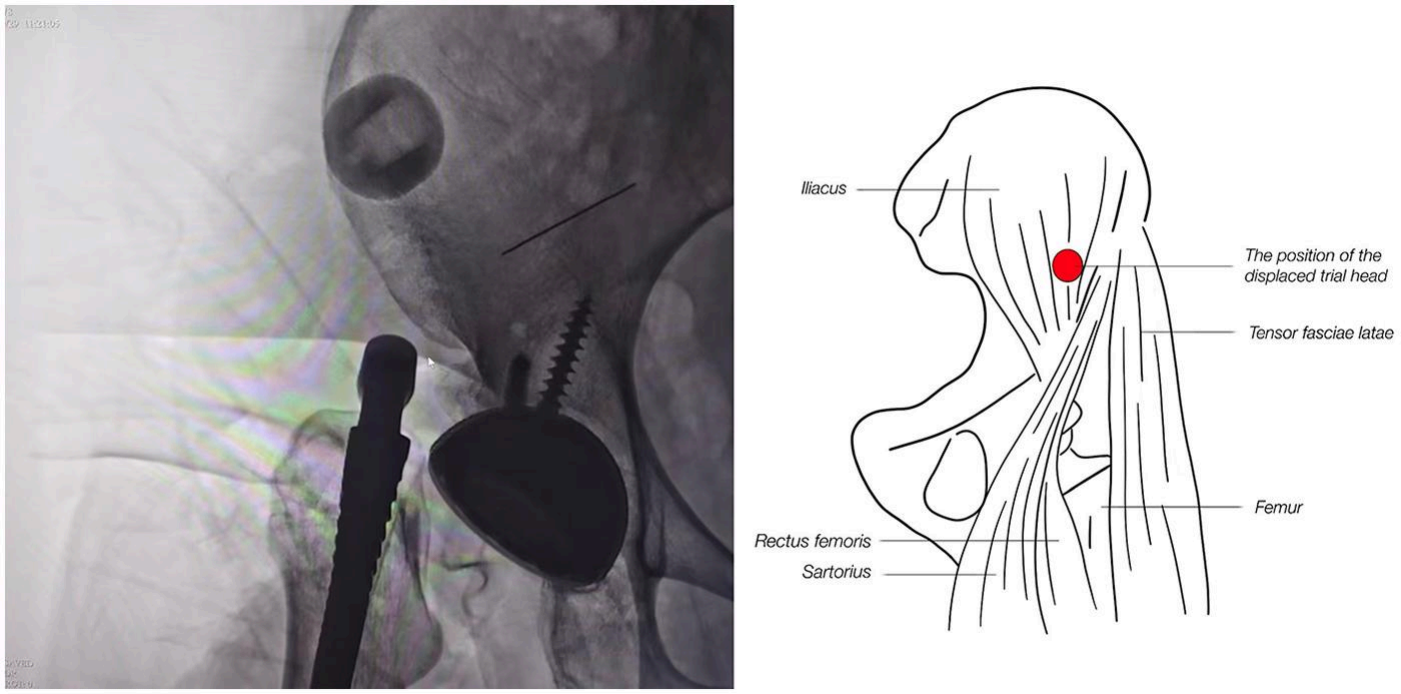
The intraoperative x-ray showed that the trial head had entered the pelvic cavity (as the diagram showing the position of the displaced trial head).

**Figure 4:**
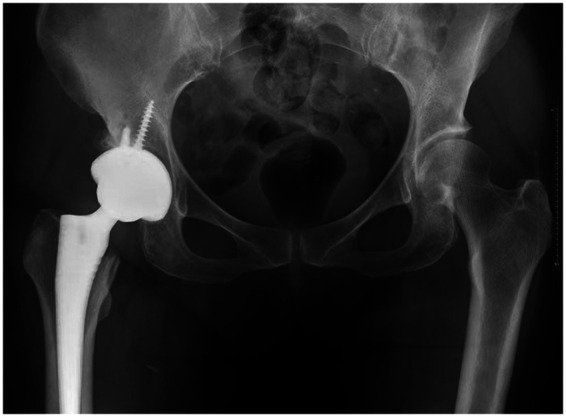
Postoperative x-ray indicated that the joint prosthesis was installed properly.

The patient was positioned supine, with the affected limb secured on a specialized surgical frame for the DAA. Surgical access was gained through the tensor fasciae latae and the sartorius muscles. After installing the acetabular and femoral components, a trial femoral head was inserted. During reduction of the hip joint while transitioning from 30° adduction-90° external rotation-40° extension (4 cm traction) to the neutral position, the trial head slipped into the interval between the rectus femoris and iliopsoas muscles, migrating into the iliopsoas plane (Figure 2). An initial retrieval attempt was made, which may have caused further displacement into the intermuscular space. Intraoperative fluoroscopy was then used to localize and retrieve the component. Although trial heads made of polymeric materials are not radiopaque, the contrast between the trial component, the surrounding bone, and the adjacent metallic trial components allowed us to identify that the trial head had migrated into the pelvis (Figure 3). After consulting with a pelvic trauma surgeon, two potential retrieval strategies were discussed. One option was transection of the rectus femoris to open the pelvic cavity. The other option was extension of the incision by the pelvic trauma team through an ilioinguinal approach. The migrated trial head appeared to be reachable through the existing anterior surgical field, whereas an ilioinguinal approach would have required a more extensive additional exposure. After weighing the need for safe retrieval against the morbidity of a larger additional approach, the former strategy was chosen to avoid further extensive additional exposure. We transected the rectus femoris muscle and turned it inferiorly, exposing the medial aspect of the iliopsoas muscle adjacent to the pubis. The trial head was finally retrieved by fingers, and the subsequent steps were completed with no incident (Figure 4). The total operative duration was 160 min, and the total blood loss was approximately 600 mL.

Postoperatively, the patient was restricted on bed with the hip and knee in a flexed position for 3 weeks to reduce tension on the repaired rectus femoris. During this period, active or resisted hip flexion, forceful straight-leg raising, rapid knee extension, and excessive stretching of the anterior thigh were avoided. Gentle isometric exercises of the peri-hip and knee muscles were performed within the patient's pain tolerance, including gluteal setting, isometric hip abduction and adduction, quadriceps setting, hamstring setting, and ankle pump exercises. Exercise intensity was increased gradually according to pain, wound healing, muscle strength, and functional recovery. The Harris Hip Score of this patient was 77 points at discharge (week 4). At the latest follow-up (May 2026), the patient had a Harris Hip Score of 83 points, with a mild pain during active elevation and no sensory disturbance was observed. Mild weakness of hip flexion was noted, with an MRC grade of 4/5, whereas knee extension strength was preserved at 5/5.

This intraoperative event also has potential medico-legal implications. Possible intraoperative complications, including soft-tissue injury and the need for additional repair procedures, should be addressed during the preoperative informed consent process. If such an event occurs, timely postoperative disclosure, accurate documentation in the medical record, and appropriate follow-up are essential. In the present case, these steps were completed to ensure transparency and continuity of care.

## Discussion

Intraoperative migration of the trial femoral head into the pelvic cavity during THA is a rare and intractable complication. It most commonly occurs during assessment of anterior stability, or dislocation after completing trial implantation (2). The mechanisms are multifactorial. Here, surgical approaches play an important role in this complication. The anterior or anterolateral approaches often require more extensive capsule and surrounding soft tissues release. In our case, a natural pathway bounded by the inguinal ligament and the rectus femoris was identified intraoperatively, through which the loosened trial head fall into the iliopsoas plane. Such unusual channel was reported previously, and was formed by relaxation of the rectus femoris while performing Hip extension and external rotation (3). What's more, anterior and anterolateral approaches often create small incisions, increasing the difficulty of retrieval due to limited exposure. Excessively medial intraoperative exposure may lead to component malpositioning. It may also create a potential space for a dislodged femoral head trial to migrate medially. In this situation, the trial head may lie closer to the medial acetabular wall, obturator region, deep aspect of the iliopsoas, or pelvic inlet. This anatomical relationship may theoretically increase the risk of intrapelvic migration (4). Some studies indicate that increased soft-tissue tension associated with these approaches may further increase the risk of intraoperative dislocation of the trial femoral head (5–7).

Currently, there is no consensus on the optimal management of such trial femoral head dislocation. Though immediate retrieval may be considered first, blind exploration or expending surgical exposure may substantially increase the risk of vascular, neural, or pelvic organ injury. Many important tissue are located close to the proximal end of the incision. If the trial femoral head penetrates too deeply into the medial side of the ilium, there is a greater possibility of damaging the external iliac artery and the femoral nerve, resulting in serious consequences (8, 9). Some reports have suggested that, for asymptomatic patients, leaving the displaced trial head *in situ*, with rigorous postoperative follow-up may represent a safe and reasonable strategy (10–13). However, in the long term, these patients may develop foreign-body reactions such as pain, restricted joint motion, peritoneal irritation signs, and prosthetic-related infection. Further migration of the trail head may result in compression, which often require revision (2, 11).

More reports opted for immediate intraoperative retrieval or prompt revision. When the trial femoral head is displaced into a relatively superficial and easily accessible area, it can be retrieved manually or with the assistance of instruments such as vascular forceps after extending incision (12, 14). When the trial head is positioned too deeply, blind exploration is inadvisable, because the standard direct anterior approach follows an intermuscular and internervous interval, and deviation from this interval may endanger adjacent structures (4). In most cases, setup of a new approach are commonly used, including ilioinguinal approach (5, 11, 15, 16), anterior iliac wing approach (17), and posterior exposure achieved by transecting the short external rotators and posterior capsule (15, 18). These techniques allow direct visualization and effective removal of the displaced trial head. However, they are technically demanding, often require additional surgical exposure, and carry a potential risk of neurovascular injury, which may limit their routine use. For extended exposure and reliable protection of intrapelvic structures, laparotomy is a definitive option for complex cases. However, such approach is highly invasive and associated with greater morbidity, longer hospitalization, and delayed recovery, therefore are often reserved as last resort (19). With advancements in medical devices, minimally invasive techniques become more popular. Laparoscopy (17, 20) provides excellent visualization of intrapelvic anatomy with reduced surgical trauma compared with open approaches. Ultrasound guidance (21) facilitated a targeted, which depends on accurate localization and favorable anatomy, minimally invasive approach via an iliac crest incision for retrieval of the migrated trial femoral head from the iliacus muscle in selected cases. These techniques enable accurate identification of the anatomical relationship between the dislodged trial head and adjacent critical structures, thereby minimizing the risk of iatrogenic injury. They also facilitate precise localization through a minimally invasive approach, potentially reducing retrieval time, postoperative pain, blood loss, and hospital stay. However, their use depends on specialized equipment and technical expertise or multidisciplinary collaboration, which may delay secondary intervention and increase procedural complexity.

In this case, retrieval of the dislodged trial femoral head by transecting the rectus femoris represents a convenient and effective way. Based on the anatomical pathway described by Sandiford, the rectus femoris constitutes the anterior wall of this channel. The transection of rectus femoris markedly improves exposure of the anteromedial acetabulum and pelvic inlet, which facilitates direct visualization for locating and removing the trial head, avoiding the risks of tissue injury. Compared with other additional extraperitoneal or intraperitoneal approaches, this method utilizes the original surgical incision, with no additional trauma and therefore shorter operative time. But it also has inherent limitations. The rectus femoris is a key biarticular muscle spanning both the hip and knee joints. Potential functional consequences include weakness in active hip flexion, difficulty with straight-leg raising, reduced quadriceps strength, impaired stair-climbing ability, and delayed functional recovery. Furthermore, expansion of anterior soft-tissue release itself may increase the risks of intraoperative bleeding, postoperative scar formation, and adhesions, potentially requiring a longer rehabilitation period. On the other side, knee extension is also generated by the vastus medialis, vastus lateralis, and vastus intermedius. Therefore, isolated injury to the rectus femoris does not necessarily result in permanent loss of knee extension strength if the muscle is repaired and protected during rehabilitation. Evidence from rectus femoris rupture repair and distal rectus femoris tenotomy suggests that knee extension strength may recover or be preserved in selected patients (22–24). However, these data are not directly derived from intraoperative rectus femoris transection during total hip arthroplasty. Focused follow-up of hip flexion strength, knee extension strength, straight-leg raising ability, gait, and stair-climbing function is therefore warranted.

We suggest transection of the rectus femoris may be considered when the dislodged trial femoral head is located along the iliopsoas and remains accessible through an extended anterior exposure, as confirmed by intraoperative imaging. It is intended to enable controlled retrieval during the index procedure while avoiding blind exploration or more invasive access. This approach is suitable for patients with moderate functional demands and intact preoperative quadriceps function, especially for individuals without neuromuscular disease or significant knee pathology who are expected to tolerate transient reductions in hip flexion and knee extension strength. For patients with high functional requirements or compromised extensor mechanisms, alternative retrieval strategies may be preferable. This case also highlights the importance of individualized decision-making. Factors such as the location of the migrated component, the surgeon's experience, and the patient's intraoperative condition should be considered when selecting the optimal management strategy. It should be noted that this retrieval method should not be considered universally applicable to all surgical approaches. In this case, it was used as a salvage option under the specific anatomical conditions of the direct anterior approach. For other approaches, the retrieval strategy should be individualized according to the location of the migrated trial head, the original exposure, and the safest access route.

Nevertheless, regardless of the abovementioned methods available, patients are inevitably subjected to varying degrees of additional injury. Preventive measures should be emphasized during surgery, including ensuring secure fixation between the trial head and the trial stem during trialing, avoiding excessive traction or forceful levering maneuvers, and routinely using a safety suture or tether.

## Conclusion

Intrapelvic migration of a trial head during total hip arthroplasty is rare but may lead to serious complications. This case highlights the importance of prompt recognition, avoidance of blind exploration, and careful assessment of the migrated trial head. Intraoperative imaging and multidisciplinary assistance may be considered when there is concern about injury to pelvic vessels, nerves, or organs. Although secure engagement of the trial head, gentle manipulation, and adequate soft-tissue protection may help reduce the risk, the mechanism and optimal management of this complication remain poorly understood. Therefore, further reports and larger clinical experience are needed to better define the risk factors, optimal management, and outcomes of this rare complication.

## Data Availability

The original contributions presented in the study are included in the article/Supplementary Material, further inquiries can be directed to the corresponding author.

## References

[1] Barton KI Steiner NJ Boldt KR Sogbein OA Tsioros SM Somerville L. Major complications after total hip arthroplasty with the direct anterior approach at a high-volume Ontario tertiary care centre. Can J Surg. (2023) 66(6):E596–601. 10.1503/cjs.005223 38056903 PMC10713201

[2] Siddiqi A Talmo CT Bono JV. Intraoperative femoral head dislodgement during total hip arthroplasty: a report of four cases. Arthroplast Today. (2018) 4(1):44–50. 10.1016/j.artd.2017.08.002 29560395 PMC5859736

[3] Sandiford NA Jack CM Rajaratnam S Weitzel S Tsitskaris K Lawrence-Watt DJ. The surgical anatomy of intra-abdominal prosthetic dislocation during hip arthroplasty. Hip Int. (2012) 22(2):184–8. 10.5301/HIP.2012.9228 22547384

[4] Geraci G Di Martino A Stefanini N Brunello M Ruta F Pilla F. Should we be concerned when the anterior approach to the hip goes accidentally medial? A retrospective study. Arthroplasty. (2024) 6(1):47. 10.1186/s42836-024-00269-9 39217376 PMC11366135

[5] Ziv YB Backstein D Safir O Kosashvili Y. Intraoperative dislocation of a trial femoral head into the pelvis during total hip arthroplasty. Can J Surg. (2008) 51(3):E73–4. 18682763 PMC2496603

[6] Takao M Nishii T Sakai T Sugano N. Postoperative limb-offset discrepancy notably affects soft-tissue tension in total hip arthroplasty. J Bone Joint Surg Am. (2016) 98(18):1548–54. 10.2106/JBJS.15.01073 27655982

[7] Higa M Tanino H Ito H Banks SA. Soft-tissue tension during total hip arthroplasty measured in four patients and predicted using a musculoskeletal model. J Exp Orthop. (2023) 10(1):130. 10.1186/s40634-023-00689-7 38051361 PMC10697917

[8] Burlage E Gerbers JG Geelkerken BRH Verra WC. External iliac artery injury following total hip arthroplasty via the direct anterior approach-a case report. Acta Orthop. (2020) 91(4):485–8. 10.1080/17453674.2020.1748287 32237979 PMC8023906

[9] Bianco Prevot L Bolcato V Bambagiotti G Tronconi LP Basile G. Nerve injury after total hip arthroplasty: etiology, preventive strategies and medico-legal considerations. Ann Med. (2025) 57(1):2600123. 10.1080/07853890.2025.2600123 41369242 PMC12697268

[10] Vertelis A Vertelis L Tarasevicius S. Trial femoral head loss in to the soft tissues of pelvis during primary total hip replacement: a case report. Cases J. (2008) 1(1):151. 10.1186/1757-1626-1-151 18789144 PMC2553055

[11] Callaghan JJ McAndrew C Boese CK Forest E. Intrapelvic migration of the trial femoral head during total hip arthroplasty: is retrieval necessary? A report of four cases. Iowa Orthop J. (2006) 26:60–2. 16789451 PMC1888581

[12] Ikeuchi K Hasegawa Y Warashina H Seki T. Intraoperative migration of the trial femoral head into the pelvis during total hip arthroplasty–report of two cases. Nagoya J Med Sci. (2014) 76(1-2):203–10. 25130007 PMC4345738

[13] Wallis C Ninomiya JT. Intrapelvic loss of a trial femoral head during total hip arthroplasty: a case report. JBJS Case Connect. (2020) 10(3):e19.00118. 10.2106/JBJS.CC.19.00118 32910582

[14] Madsen WY Mitchell BS Kates SL. Successful intraoperative retrieval of dislocated femoral trial head during total hip arthroplasty. J Arthroplasty. (2012) 27(5):820.e9–11. 10.1016/j.arth.2011.08.006 21964233

[15] Hamoui M Larbi A Delannis Y Roche O Fauré P Canovas F. Pitfall in total hip arthroplasty: intraoperative migration of the trial femoral head through the iliopsoas muscle. Eur J Orthop Surg Traumatol. (2012) 22(8):713–6. 10.1007/s00590-011-0802-1 27526075

[16] Rachbauer F Nogler M Krismer M Moritz M. Intraoperative migration of the trial femoral head into the pelvis during total hip arthroplasty: prevention and retrieval. J Bone Joint Surg Am. (2002) 84(5):881–2; author reply 2. 10.2106/00004623-200205000-00051 12013049

[17] Pita S Pato T Dos Santos AF. Intrapelvic migration of prosthetic femoral head after revision total hip arthroplasty—a case report. J Orthop Case Rep. (2022) 12(9):78–83. 10.13107/jocr.2022.v12.i09.3326 36873336 PMC9983394

[18] Kalra K Ries MD Bozic KJ. Intrapelvic displacement of a trial femoral head during total hip arthroplasty and a method to retrieve it. J Arthroplasty. (2011) 26(2):338.e21–3. 10.1016/j.arth.2009.12.005 20206465

[19] Citak M Klatte TO Zahar A Day K Kendoff D Gehrke T. Intrapelvic dislocation of a femoral trial head during primary total hip arthroplasty requiring laparotomy for retrieval. Open Orthop J. (2013) 7:169–71. 10.2174/1874325001307010169 23730381 PMC3664452

[20] Ciciriello S Gerosa M Ghezzi R Sogni A Incalza F Guttadauro A. Transperitoneal laparoscopic retrievement of a migrated prosthetic head after total hip arthroplasty: a case report. Front Surg. (2023) 10:1227026. 10.3389/fsurg.2023.1227026 37576923 PMC10413258

[21] Jiao K Ankam A. Ultrasound-assisted surgical retrieval of the lost femoral trial head during total hip arthroplasty. Can J Anaesth. (2019) 66(12):1520–1. 10.1007/s12630-019-01463-z 31485956

[22] Drefus LC Buckland MA Backus SI Root L. The functional effect of a distal rectus femoris tenotomy in adults with cerebral palsy. Gait Posture. (2014) 40(1):145–9. 10.1016/j.gaitpost.2014.03.017 24742707

[23] Salzman A Torburn L Perry J. Contribution of rectus femoris and vasti to knee extension. An electromyographic study. Clin Orthop Relat Res. (1993) (290):236–43. 8472454

[24] Hinz M Geyer S Winden F Braunsperger A Kreuzpointner F Kleim BD. Midterm outcome and strength assessment after proximal rectus femoris refixation in athletes. Arch Orthop Trauma Surg. (2022) 142(9):2263–70. 10.1007/s00402-021-04189-0 34664130 PMC8522542

